# Effects of informal learner handover in clinical dental education

**DOI:** 10.1186/s12909-023-04318-w

**Published:** 2023-05-16

**Authors:** Mehvish Shahid, Rehan Ahmad Khan, Mahwish Arooj

**Affiliations:** 1 Department of Medical Education, Central Park Medical College, Lahore, Pakistan; 2 Department of Surgery, Islamic International Medical College, Riphah International University, Islamabad, Pakistan; 3 University College of Medicine & Dentistry, University of Lahore, Lahore, Pakistan; 4 Department of Medical Education, University of Lahore, Lahore, Pakistan

**Keywords:** Learner handover, Informal learner handover, Forward feeding, Dental clinical training, Undergraduate

## Abstract

**Background:**

It is currently under discussion whether Learner Handovers (LH) are beneficial, disadvantageous, or useful in Health Professions Education. Research has not been conducted to determine the extent of existing informal learner handover (ILH) through faculty discussions. In addition to providing stakeholders with added context, examining the nature of ILH may also provide insight into the bias associated with Learner Handover.

**Methods:**

Transcripts from a series of semi-structured Focus Group Discussions (FGDs) and interviews (from January to March 2022) were iteratively reviewed to identify relevant patterns and correlations. The study involved the voluntary participation of 16 active clinical dental faculty members with a variety of designations. We did not discard any opinions.

**Results:**

It was found that ILH had a mild impact on students' training. ILH effects can be categorized into four key areas: (1) faculty behavior with students, (2) faculty expectations from students, 3) teaching approach, and 4) faculty feedback practices. Furthermore, five additional factors were identified as having a greater influence on ILH practices.

**Conclusions:**

In clinical dental training, ILH has a minor effect on faculty-student interactions. Faculty perceptions and ILH are strongly influenced by other factors contributing to the student's 'academic reputation. As a result, student-faculty interactions are never free of prior influences, so stakeholders need to take them into consideration when creating a formal LH.

**Supplementary Information:**

The online version contains supplementary material available at 10.1186/s12909-023-04318-w.

## Background

Learner Handover (LH) is the act of collecting data about various aspects of student performance and highlighting their strengths and weaknesses so that it can be presented to future teachers as an added academic aid. It has recently received attention considering its application in Competency-Based Medical Education (CBME) as a possible method of keeping a record of competencies acquired and providing teaching guidance for upcoming faculty [1–4]. Two main methods of conducting such handovers include the CLASS system proposed by Warm et al. [5] and the Milestone-Based approach tested by Schiller et al. [6] and Morgan et al. [7]

There is an ongoing debate about the LH Protocol's benefits, disadvantages, and usefulness. Since LH protocols are in development and not widely implemented, definitive data is lacking. LH advocates focus on the perceived benefits of faculty preparedness and tailoring training to match the learner. The expected result is a student who will only be assigned to work at the level of responsibility they are competent in. This will improve patient safety in the long run [1–3, 5, 8].

On the other hand, there are concerns about increased resource requirements, faculty training, and confidentiality maintenance. The most cited 'cons' include the tendency to influence future teachers’ perceptions of students. This is done by ‘setting’ the student’s educational journey based on initial impressions [1–3, 9–11]. This assimilation effect would undermine any positive intentions that may have prompted the use of the LH.

The ability of LH to bias future teachers has shown up in multiple studies [9, 12, 13]. However, the exact nature of such a bias seems disputed as different studies report different effects. These studies also tend to follow the methodological trend of testing bias in assessment by faculty members immediately after being shown the student’s earlier performance immediately before scoring [9, 11, 13]. Therefore, how student information influences faculty interaction and instruction is still unclear.

Moreover, Humphrey-Murto et al. [11] noted that faculty often engage in discussions regarding their students among their peers for various reasons. Therefore, this ‘Informal Learner Handover’ (ILH) may already influence teachers who have yet to interact with the students being discussed. This possibility has gone unaddressed and there is no research available on its propagation.

Moreover, LH Protocols can be especially useful in the lab-to-clinic transition in undergraduate dental training due to the build-up of skills required and a small, easier-to-manage student body [14, 15]. However, literature search revealed that despite the potential benefits and suitability of dental clinical training, no research explored the usage of LH in dental training.

Exploring the nature of ILH may provide added context for stakeholders about the most cited arguments against LH protocols. Furthermore, it could also offer insight into LH bias. This is so that measures can be taken when designing a Formal Learner Handover (FLH) Protocol to minimize bias propagation. Therefore, our primary goal was to explore how dental faculty members are affected by ILH practices during undergraduate dental students' clinical training. We also wanted to see how a student’s reputation among faculty affected their clinical training.

## Methods

### Study design

Semi-structured Focus Group Discussions (FGDs) were primarily used for data collection. Since the aim of the research was to explore the influence of ILH on faculty-student interactions during Dental Clinical Training, an interview guide (attached as Additional File 1) was developed to guide the flow of questions. Individual interviews were taken where participants opted out of the FGDs or when any specific point required further elaboration or exploration. This was done to encourage candid discussion and eliminate the effect of power dynamics from affecting the content of discussion. Thematic analysis using a constant comparative approach was selected to analyze the qualitative data. Ethical approval was provided by the Ethics Review Board (ERB) Committee of University College of Medicine and Dentistry, University of Lahore.

### Sampling

All full-time clinical dental faculty (*n* = 49) from two dental colleges in Pakistan were invited to take part in the study. A total of 16 faculty members volunteered to participate in the research.

The participants included all levels of teaching faculty (Professors 13%, Assistant Professors 56%, Senior Registrars 13%, and Registrars 19%). There was also no restriction placed on teaching experience. Seven departments were represented: Periodontology (31%), Oral Medicine (19%), Pediatric Dentistry (19%), Implantology (13%), Operative Dentistry (6%), Orthodontics (6%), and Endodontics (6%). There was no representation from Oral and Maxillofacial Surgery (OMFS). However, we feel that there was adequate professional diversity among the participants to provide diverse perspectives, especially since Implantology often overlapped with OMFS.

All prospective participants were sent an invitation email and WhatsApp text message with a brief introduction and a link to a Google Form that would provide details of the research and their expected role. It would also collect demographic data and record their consent to being part of the study.

### Data collection

MS conducted FGDs and interviews using Zoom Video-conferencing Software for accessibility. The date and time were arranged beforehand via WhatsApp groups for communication. Participants were asked to share their experience of employing ILH in clinical rotations they oversaw. A pre-determined Interview Guide (Additional File 1) as a general guideline to help the discussion flow. Since dental clinical training is structured into smaller, month-long ‘rotations’ where groups of students move from one department to another, inter-rotational ILH was also explored.

**Fig. 1:**
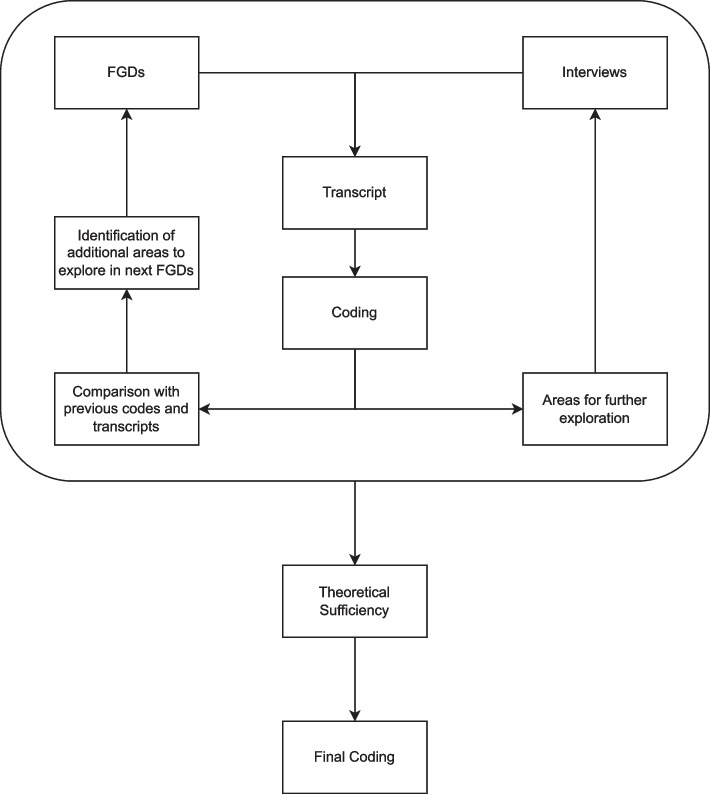
Data collection using the constant comparative approach

Each session was recorded, and the contents were transcribed verbatim using Otter.ai. Each transcript was reviewed for accuracy by MS and RAK prior to analysis and non-verbal cues were added. The overall process of data collection has been summarized in Fig. 1.

The participants were asked to primarily use the English language during the discussion to reduce time and increase transcription accuracy. However, as this limited the range of expression for certain participants who lacked proficiency, minor allowance was given for mixing Urdu and English during the discussion. MS 'spot translated' these sporadic instances during the review phase.

To ensure the anonymity of the participants and the people discussed, all identifiers were coded, and no names or personal details were included in the study. However, participants knew other participants in their own focus groups. Other participants were not informed about which participants were selected for Individual Interviews from their Focus Group.

Generally, it was noted that the participants of a junior designation (Demonstrators, Senior Registrars, etc.) seemed to be more comfortable and candid in their responses during individual interviews. The few senior faculty members (Professors and Associate Professors) selected for interviews provided similar responses to their FGDs and often spoke less in interviews. Therefore, interviews became a go-to for further probing of any interesting opinion voiced by a junior faculty member or to encourage discussion from a participant who did not share much during the FGDs. These FGDs and interviews were conducted until theoretical saturation was achieved and no new information emerged.

### Data analysis

The transcripts of each Interview and FGD were reviewed for emergent themes after they were conducted, as suggested by Saldana [16]. ATLAS.ti version 9 software was used for initial coding to identify relevant opinions and comments of interest primarily by MS. Since most of the interview revolved around dental clinical work and was jargon-heavy, MS's dentistry background was helpful in supplying context and reasoning. These were then organized into groups based on the similarity of opinion. Then, these selected opinions were reviewed and coded into categorical themes by group consensus of all authors.

Using an iterative process, the themes and codes were reviewed and modified after each added transcript. Any points of interest were identified for further exploration or incorporation into the next group discussion. Older transcripts and codes were compared with newer ones to corroborate or highlight dissent. Similar codes were compared to determine the overall scope of the category. All categorized themes and subthemes were also ‘networked’ to find interrelations by MS and reviewed by RAK and MA. Each subtheme was used as part of the overall puzzle and no opinion was discarded.

## Results

Overall, four key areas were identified that showed some effect due to ILH: (1) Faculty behavior with students, (2) Faculty expectations from students, (3) Teaching approach, and (4) Faculty feedback practices.

### Faculty behaviour with students

It was proven early on that all faculty had heard about their students in one way or another prior to teaching them. “Yes, you hear a lot about students but, as a teacher, you know. You can tell if someone is actually hardworking or not. It shows, you know, in their grades and their attitude.” – P7. The student’s past grades and attendance records were often used as a reliable indicator of how ‘serious’ a student they were. However, when further enquired about incoming students they previously heard about, almost all participants stated that their behavior towards their clinical rotation did not change between rotations. This was because they liked to assess the student’s clinical competency themselves:

“We don't go by hearsay; we prefer personal one to one experience to hearsay.” -P5.

“Because when they're coming to your…your field, then it's something completely different. And then how they will actually express themselves in that environment is going to be completely independent of whatever you've heard about them.” – P4.

There was also a unanimous sentiment that a “refresher always helps” (P4) and that it was acceptable to dedicate a week out of the clinical rotations to assess the student’s clinical ability in these ‘Refresher Weeks’. This ‘Refresher Week’ was a standard practice among clinical departments wherein they dedicated one week out of their month-long clinical rotation to evaluating their students. The reasoning given for this was that the faculty did not expect students to remember and carry over skills learned in previous years, including the year before.

As such, ILH was mostly restricted to intra-departmental discussions revolving around issues highlighted by the students themselves. Only three faculty members admitted to contacting their friends or acquaintances in other departments to get feedback on student behavior or performance. The reasoning behind this was stated as a lack of “…understanding that this is something that is required, or recognition that this is something that should be done.” (P13) In fact, when asked if they contacted faculty members in other departments, most stated patient-related instances and mentioned how they don’t consider contacting other departments for student performance or behavior feedback because of a lack of curricular integration.

This showed a distinct passivity in most faculty members who were content with letting things be unless something was specifically pointed out to them, or it was an institutional-mandated requirement. The few participants who mentioned reaching out to other departments were described as ‘friendly’ and had a strong social network at the institute. They were also often the ones who would refer to their students by name when discussing instances of events during the interviews.

This also brought to light the fact that other factors were affecting the faculty’s behavior more than the information received via ILH. Overall, 5 additional factors were identified that impacted faculty ILH practices (summarized in Table 1). It became clear that teachers needed an active institutional policy to incentivize faculty to seek constructive information about their students. It was also seen that faculty were more likely to accept ILH from someone of authority or someone in their own friends' circle. General ILH was often dismissed as gossip and student grades and performance affected its credibility.

**Table 1:** Additional factors identified that affect faculty behaviour and attitudes towards students

	**Factor**	**Finding**	**Quotes**
1	Institutional Policy	The institutional stance on which documents are shared, and when, influence faculty attitude. Unless educational collaboration is encouraged amongst departments, faculty interaction is limited to their own departments.	“I think the biggest barrier would probably be the understanding that this is something that is required, or recognition that this is something that should be done.” – P13 “No, we aren’t required to share rotation performance with anyone. I don’t think it’s allowed, basically.” – P15
2	Student Grades and attendance	Formative assessment scoring and quality of attendance have a higher impact in the faculty’s expectations and perceptions of students’ performance.	“So, as I told you, there are certain checklists and logbooks if anybody's doing the good work and get good grades on the logbooks and checklists, so we assign the patients to them” – P2 “Then there is a lot of students who are in third year, who have gone to final year, and we know that they are not interested in study, they don't come to University, and they are non-serious.” – P1
3	Faculty workload	Faculty members with extensive workload are reluctant to seek out feedback regarding student performance or divert time and energy to individually cater to student needs.	“I don’t have time for that. Going for each student, no. I cannot do that individually, if I see that a group is going to a problem, I try to, you know, try to remove the problem as a group.” – P1
4	Cultural dictations of Social and Professional Hierarchy	There is hesitancy in providing feedback to faculty members deemed higher in the institutional hierarchy due to cultural concept of respect and seniority.	“It is meant to be given to someone less skilled. So, people think you see yourself better than them if you give them feedback – especially if they are, you know, AP or Professor level.” – P15
5	Personal Bias	The personal biases harboured by faculty members influences their expectations and behaviour.	“COVID students tend to be slow. They spent so much time taking it easy, so now they find workload too much.” – P10

### Faculty expectations from students

Faculty had dichotomous expectations of their students. On the one hand, most of them mentioned how they aimed to teach their students skills that would carry over to their dental practice after graduation. On the other hand, the same faculty members also repeatedly claimed that final year students were novices when it came to patient handling.

“…there are the competence level, what we are following one is the observed status. One is the assistant status. And other one is the dependent status, students are still on the observe, assistant, and dependent status, not the independence.” – P9.

“We expect that they will be competent enough to handle the patients” – P9.

The bulk of this dichotomy seems to stem from pre-formed opinions of experienced faculty who feel that the student is good enough to be considered patient-worthy but not yet good enough to be considered a peer after graduation.

However, knowledge of tasks in earlier rotations helped faculty select which tasks to stress in their own rotations. Even though none of the institutes allowed faculty to be privy to the clinical evaluation of the students specifically before the end of the year, they do have certain expectations associated with the tasks they assume the students would have performed.

"Like, I know they have done history taking and examination so…because history taking will also be repeated by us, so I don't do to that extent because I already know that this batch, this group has already done this, so it helps in these things but not much more than that.” – P12.

As far as individual students are concerned, only high achievers and exceptionally low achievers were identified by inter-faculty discussions.

“…because discussions, a lot of discussions going on we know about a lot of students who are in the what do you say, limelight or even, even as a student, if someone is believing that he's a good student or a bad we generally know about them.” – P1.

However, they unanimously agreed that they prefer making the final judgement based on the performance of the students in their own department.

Interestingly, multiple participants mentioned outlying factors that influenced their expectations more than what they heard from each other. A common example was the repeated reference to students being “COVID batches” who studied in the “COVID era” and therefore were expected to have poor clinical and patient management skills. The general belief was that the students were inattentive in online classes and coasted through examinations designed to be easier to pass. Some participants were of the view that only theoretical knowledge had been transferred – which was insufficient for dental practitioners.

### Teaching approach

The participants’ approach to instructing their students appeared to be somewhat affected by inter-faculty discussions. The two most common points of discussion were the use of “refreshers” to recall or assess the student’s earlier learning and the management of student performance. The “Refresher Week” was a universal standard in every department, even though it took out one-third of the time allotted to their department’s clinical rotations.

“…first week is the initial evaluation and then we keep on grouping how they are working and who is good with that.” – P7.

“So, we have to repeat certain steps and clinical knowledge again, so kind of, we waste our time on that and then we go on…rather than actually going on to the patients.” – P5.

Despite some participants finding the “refresher week” a waste of time, the majority thought of it as a necessary step to gauging the student’s performance level before handing over actual patients. However, most participants also agreed that most basic skills were transferrable between departments.

“All of them are transferred. Not just one of them. Probably not to the same extent but all of them are transferrable. Facial nerve's examination for example, probably something that they can use in the Surgery Department, even Operative Department—wherever they use Local Anesthetic.” – P9.

Faculty of departments situated close by often mentioned how it was easier to coordinate teaching with each other for various activities, clinical training being one of them.

“We have the advantage…advantage that the pediatrics department and operative and endo we are under one roof, right? Yeah, the departments are different, the subject are different. So, what we do actually, we coordinate with each other, the facilitator, and the heads, we coordinate with each other.” – P4.

Even though there is no formal precedent or policy for this coordination, having departments located nearby encouraged faculty to ‘compare notes’ with each other. However, no major modifications were made to the teaching plan based on inter-faculty discussions. At most, only comments on performance or patients were exchanged. Only major institution-level directives prompted faculty to modify rotations. An example of this was the COVID-19 pandemic:

“We have modified our teaching program actually, since last one year. Before the COVID, it was only on lecture based, but we have also incorporated small group learning in our curriculum… [created batches] for the maximum facilitation during tutorials. So…we have modified our learning program, and teaching program, and since last one year.” – P2.

The reason for this stems from the expectation that students had zero exposure to patients during the pandemic. This is because the institute did not have any virtual patients ready. Therefore, the effort shifted to providing theoretical knowledge during online classes and instructional videos on dealing with patients.

However, faculty members did implement minor changes to teaching practices based on indirect information. Interfaculty discussion aided in identification of high achievers, primarily due to their class participation and co-curricular involvement. Most participants mentioned using high achievers to motivate low achievers by pairing them together.

“Like if anybody's not taking interest in doing patients so we assign the students as assistant we actually add a system to those students who are working on patients.” – P7.

However, when probed, they admitted that such a pairing only lasts for part of the rotation since they prefer finding these students by evaluating their performance in person, after discussions with colleagues in the same department. A common reason stated for this is the expectation that every student behaves differently in different departments due to their inherent preferences for different specialties.

Therefore, unless a student has been consistently performing above or below average, they do not consider taking any pre-emptive measures to tailor their training plan. However, faculty had no means of measuring this “consistent performance” apart from the student’s theory-based academic history, class attendance, and general reputation as a diligent student. Even though none of these have a direct bearing on the clinical competence of the student, participants were confident in their generalization of a good academic also being a good clinician.

### Faculty feedback practices

Feedback practices were generally uncommon among faculty. The consensus on supplying feedback to students and faculty was that it was “…not happening as often as it should” (P13). The faculty found it impractical to isolate individuals due to their overall workload and preferred targeting issues faced by most of the students.

“I cannot do that individually, if I see that a group is going to a problem, I try to, you know, try to remove the problem as a group.” – P1.

Despite having logbooks that use checklists for different skills and competencies, most departments do not look at the performance of students in other departments. They mentioned that it was not something that occurred to them. Nor did they ever consider providing other departments with feedback on the overall performance of the group. They did agree that this sort of activity would help each department’s training practices. However, some participants mentioned how such an activity could be taken as criticism and may affect inter-departmental relations. This stems from the cultural understanding that feedback “is meant to be given to someone less skilled. So, people think you see yourself better than them if you give them feedback – especially if they are, you know, AP or Professor level.” (P15) This may be why the participants agreed that while they do discuss student behavior and – to a lesser extent – performance amongst themselves, they consider it a dead-end communication “because they don't come up with a solution to all the problems discussed. Discussions occur and then we don't do anything with it. (P3)”.

This cultural tendency to include members of ‘higher faculty’ in every consideration shows up in their channels of communication as well. In response to a question about which level of faculty the participants would approach for information about the clinical prowess of the students in their batch, most agreed that the Clinical In-charge or the Demonstrators were the best judge of student performance because “demonstrators are generally more close to students and the students are more comfortable sharing their problems. (P8)” However, they all felt the need to include the respective department’s Head if the situation included patient referrals.

"The HOD of that department or the demos who are more connected to students, we will ask them for advice that 'Which student is most keen, and also a little interested in learning more work." – P4.

Therefore, it can be inferred that any actionable response does not occur without the inclusion and consent of the Head of Department.

An interesting finding that affected multiple aspects of faculty interactions was the use of ILH in managing students with some form of disability. Participants often mentioned discussing disabilities they noticed amongst their students and deciding how to help the student out in some way. The most common disability mentioned is related to speech defects like stuttering. However, some participants included students who did not speak Urdu or English as a first language in this group. The rationale for this was that these students often struggled the most when it came to dealing with patients and usually developed low confidence. Therefore, they needed extra guidance and time to develop people skills.

“Yes, there were students who had problems and the faculty, you know, told each other about them, so that they can be you know, compensated or they can be, you know, relieved, given relief of it.” P1.

Normally, students identified this way were given extra time to perform tasks.

However, the previously noted passivity also permeates the faculty’s attitude towards student learning disability or difficulty as well. Only mild learning disabilities or obvious hindrances to communication were identified by the faculty. The predominant point of view revolved around the understanding that students with severe learning disabilities would be unable to clear the rigorous and competitive admission process for medical and dental colleges. On top of that, a few participants also mentioned how they did not feel capable of recognizing most student disabilities unless the students themselves told them about them. In such cases, they did take the issue into consideration and often told their colleagues because they felt it was a more discrete way of helping the student.

## Discussion

### Informal learner handover and student-faculty interactions

Although almost none of the participants could describe a specific instance of conscious discrimination against a student based on reputation. However, all of them acknowledged knowing the reputations of high and low achievers. While some may argue that there could be subconscious biases in how they approach certain students due to the Pygmalion [17] and Golem effect [18], the fact that there is a set protocol of a refresher week followed by patient allotment in most departments, shows that their personal behavior does not particularly affect the student’s ability to procure patients for their clinical training.

However, compared to international practices noted by Cox [19], Gumuchian et al. [1], and Humphrey-Murto et al. [12], Pakistani Dental Faculty approaches student interactions passively. They do not actively address individual issues that arise with their students' clinical training unless a student approaches them first. They may discuss students with their colleagues within the same department, but it is only for minor assistance in managing students or pairing them up.

The faculty's use of ILH practices was found to be consistent with the findings by Humphrey-Murto et al. [12], who noted that faculty utilize ILH to vent frustrations among their peers. Like earlier studies, most participants mentioned that such discussions were fruitless and only a means to de-stress. The fact that no solutions were reached sometimes added to their frustrations. Therefore, it is reasonable to suggest that using their negative feedback in a formal manner may help reduce their frustrations. This would encourage them to identify areas of improvement and feel empowered to come up with solutions rather than feel discouraged and helpless.

An interesting aspect previously not explored in the literature was the coordination of faculty in consideration of students with disabilities. For instance, one of the most common issues was speech impediments. This required faculty to alter how they interacted with these students. They would give them more time and be more approachable. However, most participants expressed reservations about the idea that students with major disabilities or cognitive disorders could enter a dental program. This is due to the rigorous entrance requirements. However, previous international research has shown that 3% of medical students have some form of learning disability. 0.3% of dental students with learning disabilities are registered with their institutes to receive additional support [20, 21]. Furthermore, learning disabilities do not seem to affect exam performance [22] – which seems to be a factor around which faculty base most of their expectations in this regard.

### Factors affecting faculty handovers

Clearly, there is more to how faculty processes handover than simply being affected by what they hear. Who the ILH came from also changed how it was processed and shared. Two major factors about the source of ILH were: 1) the presence of a shared mindset and 2) the interpersonal level of comfort (friendliness) of the participants. This is seen in how participants recall approaching faculty members they are ‘friends’ with or comfortable with. This varies with the level of social interaction and the individual personality of the person involved. Also, people tend to seek out others with similar values and behaviors for their discussions, either due to Confirmation Bias [23] or Interpersonal Synchronization [24].

This may result in an opinion circulating among a select few faculty members, without long-term effects. Encouraging faculty to share information about student performance could also ease the barriers between each department. This could indirectly encourage faculty to consider different perspectives and promote collaboration. This was also clear in the discussion pattern during the FGDs. Faculty that seemed to be familiar with each other actively participated in the discussions, whereas others had to be prompted to add their perspective.

### Other factors affecting student-faculty interactions

Our research showed that, contrary to current concerns that LH would directly affect faculty expectations and assessment [1–3, 9–11, 13, 25], five other factors (Table 1) appear to play a larger role in mediating faculty behavior as well as faculty expectations and teaching methodologies. These factors can determine how LH is processed by instructors.

The passive approach to teaching seen in our research often requires explicit institutional policy to prompt action. An example of this is the case where participants did not seek feedback from other departments because the curriculum itself was not integrated. There is also reason to consider that this lack of motivation may be due to the workload clinical faculty are expected to manage. This is clear in how they prefer managing group issues rather than addressing individual issues.

Another factor not considered in current research is the professional hierarchy of faculty members and the heavy influence it has on their teaching practices. This hierarchy was one of the main reasons junior faculty hesitated to act independently. This means that the perspective of the Head of Department can potentially trickle down to the behavior of the rest of the faculty in that department. This is irrespective of any LH they receive. This was particularly clear in FGDs where junior faculty members often became silent once a professor spoke up. This often heralded the end of that discussion unless another professor carried the topic on. However, these same junior faculty members candidly shared their perspectives in interviews where they were not worried about ‘speaking over a senior'.

On top of that, faculty seems to base their expectations on test scores and pre-existing notions of the incoming batches (e.g. “COVID-batches”). Both these sources are not related to clinical performance and are unreliable.

This shows that ILH's inherent effects cannot simply be attributed to assimilation bias or hive thinking [3, 9, 11, 13]. There are many sides to how information is transferred and accepted by individuals. Therefore, it requires a deeper understanding of the environment in which handovers occur.

The development of a FLH Protocol that accounts for the subjectivity of the faculty and the institution can contribute immensely towards homogenizing students' training. If used appropriately, a well-designed handover system could be used by almost all dental institutions to provide a longitudinal outlook on the student’s journey and ability. Therefore, they can provide a way to standardizing dental graduates' competence on a national or international level [6, 26, 27]. Therefore, any FLH protocol developed would need to take this subjectivity into account and reduce the possibility of harm.

By placing importance on the Handover Protocol, it is possible to move away from the widespread practice of faculty members focusing solely on numerical assessment scores. This aligns with the global trend of investigating how Handover Reports influence student assessment scores [9–11, 13, 25]. The use of Handover Reports as a teaching aid rather than a final evaluation tool can be more beneficial since the numerical evaluation of overall performance can overlook significant performance-related details, which may not accurately reflect the clinical competence of the trainee dentist.

### Limitations

Since only faculty opinions were collected, it could be considered a one-sided narrative based on self-reported data. Similar perspectives from students could help provide additional corroboration. Also, the study was conducted in Pakistan and may not reflect international perspectives. However, the general conclusions drawn about ILH effects should still be relevant, even if the specifics are not. Since the participants had to mostly use English for their discussions, it is possible that they were unable to fully articulate their opinions. The minor allowance for Urdu helped overcome this limitation to a certain extent, however certain participants may still have found their expression limited. The voluntary nature of participation may have resulted in the inclusion of faculty with a particular mindset. Also, unconscious effects could not be deeply explored. Even though the data analysis process included all authors, MS provided the initial codes. This may have introduced a personal bias in coding despite the review and input provided by RAK and MA at other stages of analysis. A longitudinal comparison with the inclusion of feedback given to students over the course of their clinical training may further explore the ground realities of informal handover practices in clinical training.

## Conclusions

Our work sheds light on the effects of ILH currently being experienced in undergraduate clinical dental training. Sharing an opinion about a student, which can ultimately affect their career, is a considerably more nuanced process than prior studies have suggested. Simply sharing information alone does not always lead to students being treated differently. Our results showed that faculty made minor teaching modifications according to ILH received and often used it to discretely aid students with certain disabilities. It also shows that institutional culture and the personal belief system have a distinct role in how faculty treat ILH. Moreover, there are multiple factors that interact with each other to generate a localized ILH culture. Therefore, the presence of a formal protocol for LH transfer may ease departmental barriers and provide a more holistic view of a student’s performance and improvement.

## Supplementary Information

**Additional file 1.**

## Data Availability

The transcripts that support the findings of this study are available on request from the corresponding author MS. They are not publicly available due to the discussion of alternate individual identifiers that may compromise confidentiality of the participants, or the students discussed.

## Abbreviations

LH: Learner Handover
CBME: Competency Based Medical Education
MH: Milestone handover
PD: Program Director
PPI: Prior Performance Indicators
ERB: Ethical Review Board
ZPD: Zone of Proximal Development
PRISMA: Preferred Reported Items for Systemic and Meta-Analysis
