# Supplementary material for: Effects of informal learner handover in clinical dental education
Source: BMC Med Educ. 2023 May 16;23:339. doi: 10.1186/s12909-023-04318-w (PMC10186315; doi:10.1186/s12909-023-04318-w)
Supplement: Supplementary file 1 — Additional file 1. [file 12909_2023_4318_MOESM1_ESM.docx]

The Interview Guide: Questions and Prompts for Focus Group Discussions and Interviews for “Effects of Informal Learner Handover in Clinical Dental Education”

*The following questions are a general guide to aid in a smooth flow of the discussion and to make sure no major topic is overlooked. The focus group discussions and interviews followed an organic pattern where these questions may be modified or not asked entirely if covered by the participant by themselves.*

Q1. What are your expectations from your upcoming rotation?

Prompts: What makes you think this way? Why is that?

Q2. Are there any students you know about based on their general reputation?

Prompts: What do you know about them? Where did you first hear about them?

Q3. Have you ever received information about a class or student that made you consider changing your teaching strategy to suit them?

Prompts: What was the situation? How did you receive this information? How often does this happen?

Q4. How do you gauge the level of competence of your students?

Prompts: Is there any report or feedback process that you find particularly helpful?

Q5. Have you ever discussed a difficult student with a colleague?

Prompts: Why not? (If the answer is no) How did it help? (If the answer is yes.)

Q6. How do you allocate patients to students?

Follow-up: Have you ever withheld patients from students or allowed early access to patients for some students?

Prompt: Why?

Q7. Who do you think is best suited to comment on the clinical competence of a student? (Example situation: If you had to select a student from a different rotation for treatment of a personal patient, who would you approach in that department for the best suggestion?)

Prompts: Why?

Q8*. Have you ever given or been given information about a student’s disability by a different faculty member?

Prompts: Why? How did you act on it?

*(*Added later due to multiple instances of it coming up.)*
